# Domestic trauma with penile and scrotum skin degloving and testicular avulsion

**DOI:** 10.1093/jscr/rjab175

**Published:** 2021-05-26

**Authors:** M Iafrate, N Leone, M Mancini, T Prayer, F Bassetto, F Dal Moro

**Affiliations:** Urological Clinic, Department of Surgical, Oncological and Gastroenterological Sciences, University of Padua, Padua, Italy; Urological Clinic, Department of Surgical, Oncological and Gastroenterological Sciences, University of Padua, Padua, Italy; Urological Clinic, Department of Surgical, Oncological and Gastroenterological Sciences, University of Padua, Padua, Italy; Urological Clinic, Department of Surgical, Oncological and Gastroenterological Sciences, University of Padua, Padua, Italy; Plastic and Reconstructive Surgery Unit, University of Padua, Padua, Italy; Urological Clinic, Department of Surgical, Oncological and Gastroenterological Sciences, University of Padua, Padua, Italy

## Abstract

Traumatic lesions of male external genitalia are certainly less frequent than the other body sites and in the majority of cases they are caused by work accidents in the metalworking environment or by gunshot wounds. We present a rare case of traumatic degloving lesion of the male external genitalia with avulsion of the left testis caused by an accidental fall from the ladder. Reconstructive surgery was carried out in a single procedure, obtaining an excellent esthetic and functional result.

## INTRODUCTION

Skin avulsions of male genitalia are rare urological emergencies. Although they are not life threatening, they can have a strong psychological impact [1]. Generally, the lesions are limited only to the skin, with minimal bleeding and without damage to the most important structures such as the corpora cavernosa, the spongy body or the testicles. [2]

Penis and testicles generally have a lower risk of traumatic injury due to their relative isolation and mobility. Domestic trauma with penile and scrotum skin degloving and testicular avulsion is a rare condition which requires a prompt surgical solution with challenging options.

## CASE REPORT

**Figure 1:**
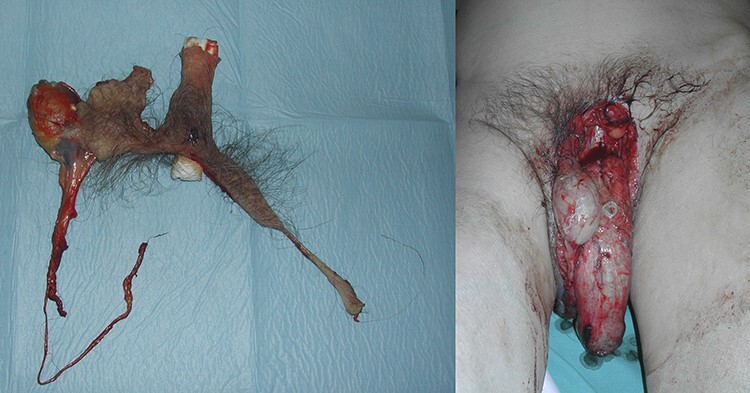
Avulsed right testis with skin shaft completely removed.

**Figure 2:**
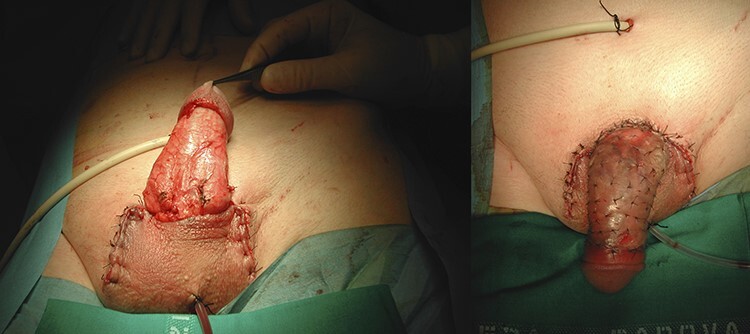
Skin graft to overlay the penile shaft through the quilting technique.

A 60-year-old male came to our emergency room after a domestic injury. The patient reported that he had slipped off the ladder while trying to reach the highest shelf of belief. During the fall he suffered an injury to his genitals. Physical examination documented the complete degloving of the pubis, the penis and scrotum without the presence of the right testis. The patient did not report pain. The skin that covered the shaft was completely detached and separated from the organ with the avulsed right testis (Fig. 1). An abdominal contrasted computed tomography scan excluded any fracture or lesion to the organs but confirmed the traumatic removal of the left testicle. Under general anesthesia, the surgical team, composed by a urologist and a plastic surgeon, performed a cleaning and debridement of the avulsed skin flap that covered the penile shaft. An epicystostomy catheter was put in place. The testis was covered with the scrotal skin attached to the perineum assuming its vitality due to the skin pedicle with an apparently good blood supply. The avulsed skin flap has been disinfected and thinned removing all the subcutaneous tissue, then positioned as a thin skin graft to cover the penile shaft through the quilting technique (Fig. 2). At the follow-up visit after 1 month the skin graft was healthy, without damage to the urinary and sexual function.

## DISCUSSION

Domestic accidents are a very rare cause of genital trauma with skin avulsion.

In the past, the most frequent causes of avulsion of the genital skin were accidents with agricultural machinery; to date, these injuries derive most commonly from motor vehicles accidents [3–5]. Other causes may be secondary to various devices such as penile rings, vacuum cleaners, industrial machine accidents or animal bites [2, 6]. Degloving injuries of the penile skin are not a painful condition [7]. After a good hemostasis on the residual funicular stump of the left testicle, the avulsed skin was used to cover the shaft with the quilting technique [8]. This technique is largely used for the mucosal grafts, and allows reduction the risk of seroma formation. Residual scrotal skin was used to reconstruct the scrotal sac. In cases where the skin is insufficient, it is possible to bury the penis in the scrotum or in the suprapubic region or use acellular dermal matrix as a first step, before using a delayed autologous skin graft. There are also other techniques involving testicular burial in the groin region or in the inner part of the thigh as well as expansion of the scrotal tissue. Several complications have been reported, such as edema, infections, hematomas, seromas and scar retraction [9].

## CONCLUSIONS

Immediate treatment of traumatic penile degloving must be as conservative as possible.

Use of the avulsed skin, when possible, with the quilting technique is the preferable choice for reconstructing the penile skin, in order to assure good cosmetic and functional results.
